# Acceptance of smart sensing, its determinants, and the efficacy of an acceptance-facilitating intervention in people with diabetes: results from a randomized controlled trial

**DOI:** 10.3389/fdgth.2024.1352762

**Published:** 2024-05-28

**Authors:** Johannes Knauer, Harald Baumeister, Andreas Schmitt, Yannik Terhorst

**Affiliations:** ^1^ Department of Clinical Psychology and Psychotherapy, Institute of Psychology and Education, University Ulm, Ulm, Germany; ^2^ Research Institute Diabetes Academy Mergentheim (FIDAM), Bad Mergentheim, Germany; ^3^ Department of Psychological Methods and Assessment, Ludwigs-Maximilian University Munich, Munich, Germany

**Keywords:** smart sensing, diabetes mellitus, digital health, acceptance, implementation, UTAUT

## Abstract

**Background:**

Mental health problems are prevalent among people with diabetes, yet often under-diagnosed. Smart sensing, utilizing passively collected digital markers through digital devices, is an innovative diagnostic approach that can support mental health screening and intervention. However, the acceptance of this technology remains unclear. Grounded on the Unified Theory of Acceptance and Use of Technology (UTAUT), this study aimed to investigate (1) the acceptance of smart sensing in a diabetes sample, (2) the determinants of acceptance, and (3) the effectiveness of an acceptance facilitating intervention (AFI).

**Methods:**

A total of *N* = 132 participants with diabetes were randomized to an intervention group (IG) or a control group (CG). The IG received a video-based AFI on smart sensing and the CG received an educational video on mindfulness. Acceptance and its potential determinants were assessed through an online questionnaire as a single post-measurement. The self-reported behavioral intention, interest in using a smart sensing application and installation of a smart sensing application were assessed as outcomes. The data were analyzed using latent structural equation modeling and t-tests.

**Results:**

The acceptance of smart sensing at baseline was average (*M *= 12.64, *SD *= 4.24) with 27.8% showing low, 40.3% moderate, and 31.9% high acceptance. Performance expectancy (*γ *= 0.64, *p *< 0.001), social influence (*γ *= 0.23, *p *= .032) and trust (*γ *= 0.27, *p *= .040) were identified as potential determinants of acceptance, explaining 84% of the variance. SEM model fit was acceptable (RMSEA = 0.073, SRMR = 0.059). The intervention did not significantly impact acceptance (*γ *= 0.25, 95%-CI: −0.16–0.65, *p *= .233), interest (OR* *= 0.76, 95% CI: 0.38–1.52, *p *= .445) or app installation rates (OR* *= 1.13, 95% CI: 0.47–2.73, *p *= .777).

**Discussion:**

The high variance in acceptance supports a need for acceptance facilitating procedures. The analyzed model supported performance expectancy, social influence, and trust as potential determinants of smart sensing acceptance; perceived benefit was the most influential factor towards acceptance. The AFI was not significant. Future research should further explore factors contributing to smart sensing acceptance and address implementation barriers.

## 1 Introduction

Diabetes emerged as one of the most serious and common chronic diseases with approximately half a billion people affected worldwide (1, 2). The metabolic disease has far-reaching implications (3–7), creating an immense burden for affected individuals as well as health care systems (2, 8). Moreover, the prevalence rates of mental health problems are significantly higher among people with diabetes (9). For instance, studies show prevalence rates of comorbid depression in people with diabetes ranging from 12%–27% (10, 11), which is considerably higher compared to the general population. Comorbid mental health problems may not only aggravate the burden associated with diabetes but also give rise to an even greater strain of affected individuals and healthcare systems (12–14). Hence, effective treatments supporting mental health are needed for this population.

Current mental health treatment options for people with diabetes range from face-to-face treatment (15–17) to digital interventions (18, 19). In order to provide optimal and effective treatment, accurate and early detection of mental problems is key. Early detection of symptoms enables affected individuals to take preventive measures and clinicians to intervene appropriately (20–22). However, due to economic constraints and limited resources in health care systems (23, 24), mental health problems often go unrecognized and untreated in general practice (25, 26), thus creating a need for innovative diagnostic methods. One such method could be smart sensing, with the goal of achieving scalable, precise, and time-effective detection of symptoms (27–29). Smart sensing refers to the passive collection of digital markers and features via smartphones and other wearables (30). Tracking usage data from mobile devices as well as built-in sensor data (e.g., GPS, accelerometer, light sensors) offers the possibility to draw conclusions about health status and behaviors of individuals (31, 32). Recent studies support the great potential in the context of mental health (33–38). For example, studies were able to distinguish between individuals with vs. without depressive symptoms based on their GPS data with an accuracy of 86% (39) and could classify depressive symptoms based on sensing variables with an accuracy of 81% (40).

In the daily lives of individuals with diabetes, sensor and tracking technologies already constitute integral components frequently (41). The continuous monitoring and self-regulation necessitated by the metabolic disorder commonly involve the prominent utilization of sensor technology and insulin pumps (42). Within this framework, smartphones take over an increasingly crucial and efficient role as an interface (43–46). Considering the existing prevalence of monitoring and sensing modalities for individuals with diabetes, this demographic may constitute a noteworthy target group for the implementation of smart sensing technologies with a focus on mental health.

However, parallel to integrating innovative diagnostic methods like smart sensing into clinical practice, it is necessary to evaluate its acceptance among people with diabetes as a prerequisite for utilization. The framework for technology acceptance and usage, known as the Unified Theory of Acceptance and Use of Technology (UTAUT) (47), has found extensive application (48, 49). The UTAUT pinpoints four factors influencing acceptance and behavioral intention. Accordingly, the fundamental determinants of acceptance are: (1) performance expectancy (perceived personal advantage gained from using the technology), (2) effort expectancy (expected simplicity of use), (3) social influence (belief that the technology is valuable to others), and (4) facilitating conditions (anticipated assistance and accessibility of tangible resources). This model could serve as a framework for exploring acceptance of smart sensing and its underlying factors. Furthermore, trust has been recognized as a pivotal factor influencing the acceptance of technology and artificial intelligence augmented systems in various application domains (50–52). For example, an individual may receive feedback from a smart sensing system indicating the detection of heightened stress levels and an increased risk of worsening mental symptoms, thus suggesting preventive measures. Depending on the trust in the system, users could either follow the recommended actions or reject them. The first studies that applied the UTAUT framework in the context of smart sensing therefore additionally assessed trust in the technology as a potential facilitator and predictor towards acceptance (53).

Following up on a previous study conducted in a healthy population (53), the present study explores the acceptance of smart sensing a population of people with diabetes to address the following question:

What is the acceptance of smart sensing in the context of mental health for people with diabetes?

Furthermore, the present study seeks to apply the extended UTAUT framework to validate this model in a diabetes sample.

We hypothesize that the UTAUT factors performance expectancy, social influence as well as the factor trust are potential determinates of acceptance of smart sensing.

To successfully implement technologies and ensure uptake and optimal use, it is essential to investigate options to promote the acceptance of said technologies. One way to influence acceptance is using acceptance facilitating interventions (AFI). With a theory-based approach (e.g., UTAUT), AFI target specific factors to influence acceptance—for example, by emphasizing personal gain (performance expectancy) or depicting positive user reports (social influence). Considering possible modalities for AFI, especially video-based AFI offer numerous benefits and have been successfully implemented in the context of blended therapy and internet-based mental health interventions (54, 55). Furthermore, with regard to the situational circumstances suitable for the deployment of AFI, optimizing the time spent in clinical waiting rooms for the implementation of video-based interventions could prove advantageous. Although, AFI have been effectively used in numerous studies (55–58), there has been limited research on the impact in the context of smart sensing as well as in a diabetes population.

Thus, the present study investigates how the acceptance of smart sensing in a diabetes sample is influenced by a UTAUT-based AFI compared to an attention control group.

We hypothesize that (a) the self-reported acceptance, (b) the interest in using a smart sensing app, and (c) the rate of actual installation of a smart sensing app on personal smartphones will be higher in the intervention group compared to the control group.

Lastly, in order to understand the acceptance of smart sensing in a more comprehensive way there are a few factors possibly influencing behavioral intention that should be paid attention to. A meta-analysis of the UTAUT (49) found education to be an important factor influencing behavioral intention (*r *= 0.18, *p *< 0.05). Furthermore, a study on personality traits as predictors of perceived and actual usage of technology (59) found significant correlations between behavioral intention to use a technology and conscientiousness (*r *= 0.15, *p *< .05) and agreeableness (*γ *= 0.29, *p *< .05). Hence, in sensitivity analysis we analyzed the correlations between behavioral intention to use smart sensing and (a) education and (b) personality.

## 2 Methods and materials

### 2.1 Study design and sample

A short-term randomized controlled trial with one measurement time point at post-treatment was conducted online to investigate the effect of the AFI. Participants were randomly assigned to either an intervention group (IG) or a control group (CG). The randomized allocation of participants was automatically managed by the online survey platform LimeSurvey. The study was approved by the Ethics Committee of Ulm University (398/21 – CL/bal.). Participants were enrolled from June 1, 2022 till December 31, 2022. Reporting on this study we follow the CONSORT guidelines (60) (see Supplementary S1).

### 2.2 Inclusion criteria and data collection procedures

The survey, including all procedures and data collection, was conducted online. Participants were recruited via an e-mail list targeting a study panel of people with diabetes diagnosis as well as study flyers. People were eligible to participate if they met the following self-reported inclusion criteria: (1) aged 18 years or older, (2) being diagnosed with diabetes, (3) having internet access, (4) providing informed consent, and (5) agreement to data processing procedures according to the European General Data Protection Regulation. If any criteria were not fulfilled, participation was rejected.

After answering socio-demographic questions, participants were randomized to either the IG or CG and watched the according video (AFI or control video). Although group allocation was not explicitly mentioned, participants were aware of two study conditions due to the informed consent. After watching the video, the acceptance of smart sensing as well as all assumed determinants were assessed. Furthermore, interest in signing up for a smart sensing study and actual installation rate of a smart sensing app was assessed.

### 2.3 Intervention and control condition

Participants in the intervention group watched a whiteboard based AFI video with a total duration of 4:34 min. The UTAUT model served as a basis for the structure and content of the video. Accordingly, the video focused on the following assumed determinants of acceptance: performance expectancy (e.g., application areas, such as self-monitoring and early recognition of mental health symptoms, personal benefits), effort expectancy (e.g., passive data collection, personal involvement), facilitating conditions (e.g., low necessary personal resources), and social influence (e.g., population-specific positive examples and user reports). Based on previous studies, the video additionally aimed to generate trust in the technology (e.g., data safety, anonymized processing). The AFI video started with a general explanation of smart sensing. Next it delved into which data can be collected via smart sensing as well as the process of data collection. The video further explored different application areas of smart sensing. Lastly, positive user experiences, tailored to a population with diabetes, were presented. A more detailed outline of the AFI can be found in Supplementary S2.

Participants in the control group watched an educational video on the concept of mindfulness, the influence of mindfulness on health, and suggestions on how to integrate mindfulness into one's daily life. The duration of the video was 3:00 min.

### 2.4 Measures and outcomes

#### 2.4.1 Participant characteristics

Demographic variables (i.e., age, gender, nationality, relationship status, and education level), personality dimensions, general mental health symptoms, and diabetes-specific mental health aspects were assessed using a set of questionnaires.

Basic personality dimensions were assessed with the 10-item version of the Big Five Inventory (BFI-10) (61). The BFI-10 evaluates openness, conscientiousness, extraversion, agreeableness, and neuroticism with a 5-point Likert scale from “fully disagree” to “fully agree”. For evaluation purposes, the mean score was computed for each subscale.

##### 2.4.1.1 Health status

Depression symptoms over the past two weeks were assessed using the 8-item Patient Health Questionnaire (PHQ-8). Using a 4-point Likert scale ranging from “Not at all” to “Nearly every day”, a sum score is computed (range 0–24). Higher scores indicate higher depressive symptoms, and scores of 10 or higher are considered to indicate clinically elevated depressive symptoms (62).

Anxiety symptoms over the last two weeks were assessed with the 7-item Generalized Anxiety Disorder Questionnaire (GAD-7). Items are answered on a 4-point Likert scale from “not at all” to “nearly every day”. Sum scores range from 0 to 21 with higher scores indicating higher anxiety symptoms (63).

Sleeping problems were assessed with the Insomnia Severity Index (ISI-7). The scale consists of seven questions regarding worries, occurrence, and severity of abnormal sleep patterns, and their harmful effects. The questions are answered on a 5-point Likert-scale ranging from 0 to 4. High sum scores (range 0–28) indicate more sleep problems (64).

The Self-Efficacy Scale (SES) assesses self-efficacy using questions on one's perceived personal competence and control. The inventory consists of 10 items answered on a 4-point Likert scale with higher scores (range 10–40) indicating higher self-efficacy (65).

The Fear of Progression Questionnaire Short Form (FoP-Q-SF) assesses worries and fear of disease progression and its consequences using 12 items and a 5-point Likert scale (from “never” to “very often”). Higher total scores (range 12–60) indicate higher fear of progression (66).

To assess the degree of possible participation and hindrance, the Index for the Assessment of Health Impairments (IMET) was used. The IMET consists of nine questions answered on a 11-point Likert scale. Item scores are summed to a total score (range 0–90) indicating the extent of impairment (67).

Emotional distress related to diabetes was assessed using the Diabetes Distress Scale (DDS). The questionnaire consists of 17 items requesting emotional problems related to diabetes in the last 4 weeks. The items can be summed to a total score as well as four subscale scores to evaluate specific levels of distress. The scale scores range from 0 to 6 with higher scores indicating higher distress (68).

The Diabetes Self-Management Questionnaire–Revised (DSMQ-R) is a 20-item battery which assesses diabetes self-care activities aiming to manage glucose levels and prevent long-term complications. For analysis of diabetes self-care, the sum score (range 0–10) was calculated (69).

#### 2.4.2 Acceptance measures

Self-reported acceptance was assessed with the behavioral intention scale of the UTAUT questionnaire (47, 49, 70). The scale consists of four items rating one's intention to use a smart sensing app on a five-point Likert scale (ranging from “fully disagree” to “fully agree”). The sum score was categorized as suggested in previous studies (54–58): low acceptance = sum scores from 4 to 9, moderate acceptance = sum scores from 10 to 15, and high acceptance = sum scores from 16 to 20. Secondly, the level of interest was gauged based on the count and proportion of participants who explicitly expressed a willingness to utilize a smart sensing app. Subsequently, the behavioral outcome was evaluated directly by examining the count and percentage of actual installations of the smart sensing app.

#### 2.4.3 Determinants of acceptance

As potential determinants of acceptance, performance expectancy (3 items), effort expectancy (3 items), social influence (2 items), and facilitating conditions (2 items) were assessed with the UTAUT questionnaire (47, 49, 70). All items were rated on a five-point Likert scale from “fully disagree” to “fully agree”. All UTAUT items can be found in Supplementary S3.

Trust in the technology was assessed with the short form of the German Automation Trust Scale, adapted to the digital health context (50, 71). Seven items are rated on a seven-point Likert scale from “fully disagree” to “fully agree”. The sum score ranges from 7 to 49 with high scores indicating high trust in the technology.

### 2.5 Statistical analysis

All data analysis followed the per-protocol principle. Participants that dropped out before the randomization or did not receive the intervention as well as were removed. Demographic, mental health, and acceptance-related variables were analyzed using standard descriptive statistics. *P*-values <0.05 were considered to indicate statistical significance in all analyses.

#### 2.5.1 Acceptance of smart sensing for health

Following previous studies on the acceptance of digital interventions (54–58), the acceptance of smart sensing was assessed as self-reported acceptance, rates of self-reported interest in using smart sensing, and actual installation rates of a smart sensing application (technologically validated via a smart sensing app). The general acceptance of smart sensing is assumed using the acceptance in the CG which did not receive any AFI.

#### 2.5.2 Predictors of acceptance: latent structural equation modeling

The influence of potential determinants of acceptance was investigated using latent structural equation modeling (SEM). A measurement model consisting of latent factors for all items of acceptance, performance expectancy, effort expectancy, facilitating conditions, social influence, and trust was defined as a first step. In the next step, the effects of the latent factors on acceptance were introduced. The proposed predictors performance expectancy, social influence and trust were tested one-sided based on the previous model on acceptance of smart sensing (53).

The root mean square error of approximation (RMSEA) as a non-centrality parameter and the standardized root mean square residual (SRMR) as a residual index were used to assess the goodness of fit (72–74). Acceptable model fit was determined using accepted cut-off guidelines for RMSEA (<0.08) and SRMR (≤0.08) (75–77). Missing date was handled using full information maximum likelihood (78). Robust (Huber-White) standard errors were obtained.

#### 2.5.3 Intervention effects

The effects on acceptance were analyzed on a dimensional level. A t-test was used for the observed data and effects on the latent level were investigated using the SEM, introducing group allocation into the model as a dummy-coded predictor.

#### 2.5.4 Sensitivity analysis

To explore the relationship between education, personality and acceptance we performed correlative sensitivity analysis. Education level (higher values indicating higher education) was summarized according to the International Standard Classification of Education: ISCED-11. Personality was investigated for each subscale (openness, conscientiousness, extraversion, agreeableness, and neuroticism).

### 2.6 Software

The statistical software R was used for all analyses (79). The R package “lavaan” was used as the core package for all structural equation models (80). See Supplementary S4 for an overview of all packages and versions used in the present analysis.

## 3 Results

**Figure 1:**
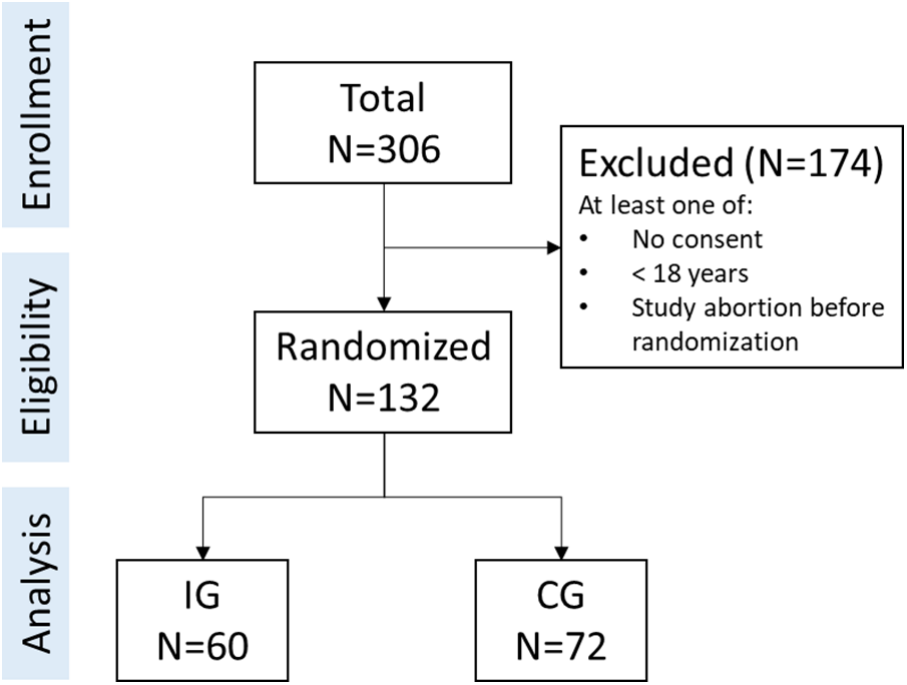
Study flow.

A total of *N* = 132 individuals provided informed consent, were included in the study, and randomized to their groups (CG: *n* = 72; IG: *n* = 60). The study flow is depicted in Figure 1. Participants were between 27 and 81 years of age (*M *= 57.63, *SD *= 12.39). Gender was distributed unequally (40.2% female, *n* = 53). Most participants (60.6%, *n* = 80) had an advanced qualification level (e.g., bachelor degree and higher). A majority had a diabetes type-1 diagnosis (74.2%, *n* = 98) compared to a diabetes type-2 diagnosis (25.8%, *n* = 34). On average, participants reported mental health symptoms below clinical relevance (PHQ-8: *M *= 5.74, *SD *= 4.57; GAD-7: *M *= 4.32, *SD *= 3.72; ISI-7: *M *= 7.60, *SD *= 5.76). Diabetes Distress was *M *= 1.71 (*SD *= 0.86), whilst the average diabetes self-management score suggested suboptimal behavior (*M *= 4.13, *SD *= 0.51). For further details and group-specific information see Table 1.

**Table 1:** Sample characteristics.

	All *N* = 132	IG *n* = 60	CG *n* = 72
Demographics			
Age, mean (SD)	57.63 (12.39)	58.23 (12.64)	57.125 (12.25)
Female gender	53 (40.2%)	26 (43.3%)	27 (37.5%)
Nationality			
German	127 (96.2%)	59 (98.3%)	68 (94.4%)
Others	5 (3.8%)	1 (1.7%)	4 (4.6%)
Relationship status			
Single	27 (20.5%)	11 (18.3%)	16 (22.2%)
In relationship	105 (79.5%)	49 (81.7%)	56 (77.8%)
Qualification level^a^			
Basic	2 (1.5%)	0 (0%)	2 (2.8%)
Intermediate	50 (37.9%)	20 (33.3%)	30 (41.7%)
Advanced	80 (60.6%)	40 (66.7%)	40 (55.6%)
Diabetes type			
Type-1	98 (74.2%)	48 (80%)	50 (69.4%)
Type-2	34 (25.8%)	12 (20%)	22 (30.6%)
Personality facets, mean (SD)			
Openness	3.28 (0.94)	3.27 (1.02)	3.29 (0.88)
Conscientiousness	2.90 (0.77)	2.86 (0.70)	2.94 (0.82)
Extraversion	3.12 (0.95)	2.96 (0.86)	3.26 (1.00)
Agreeableness	3.16 (0.79)	3.19 (0.70)	3.14 (0.87)
Neuroticism	2.78 (0.99)	2.59 (0.98)	2.95 (0.97)
Health variables, mean (SD)			
Depressive symptoms (PHQ-8)	5.74 (4.57)	5.36 (4.14)	6.08 (4.92)
Anxiety symptoms (GAD-7)	4.32 (3.72)	3.80 (3.54)	4.77 (3.85)
Sleep problems (ISI-7)	7.60 (5.76)	7.98 (5.97)	7.29 (5.61)
Diabetes distress (DDS)	1.71 (0.86)	1.66 (0.90)	1.76 (0.83)
Fear of progression (FoP-Q-SF)	24.91 (8.69)	23.78 (8.71)	25.88 (8.63)
Self-efficacy (SES)	29.73 (3.89)	30.00 (4.09)	29.50 (3.73)
Diabetes self-management (DSMQ)	4.13 (0.51)	4.23 (0.57)	4.04 (0.43)
Health impairment (IMET)	16.67 (17.12)	17.81 (16.63)	15.73 (17.62)

^a^ Education level is summarized according to the International Standard Classification of Education: ISCED-11.

### 3.1 General acceptance of smart sensing

**Figure 2:**
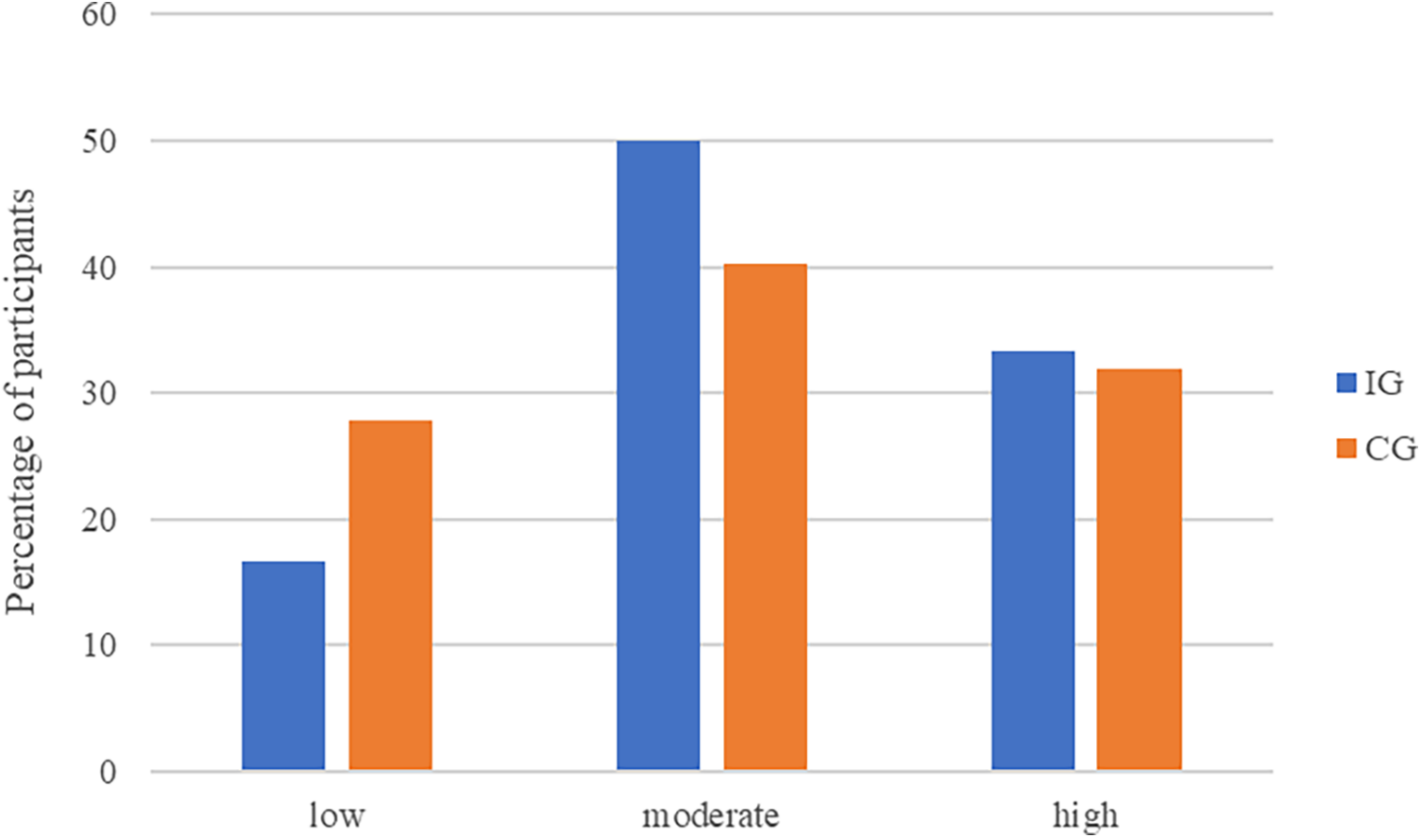
Acceptance of smart sensing levels across treatment groups. The UTAUT behavioral intention sum score categorized acceptance as low (sum score: 4–9), moderate (sum score: 10–15), and high (sum score: 16–20).

In the CG a total of *n* = 20 participants (27.8%) reported low, *n* = 29 (40.3%) moderate, and *n* = 23 (31.9%) high acceptance (see Figure 2). The unmanipulated self-reported acceptance of smart sensing in the CG was average *M *= 12.64 (*SD *= 4.24, Min = 4, Max = 19).

A total of *n* = 36 (50.0%) participants indicated interest in trying out smart sensing in another study (no interest: *n* = 16, 22.2%; not responded: *n* = 20, 27.8%). Of all 36 participants with interest, only *n* = 12 (33.3%; 16.7% of all participants in the CG) installed the smart sensing app.

### 3.2 Predictor variables associated with acceptance

The final measurement model for acceptance, performance expectancy, effort expectancy, facilitating conditions, social influence, and trust, showed an acceptable fit (RMSEA = 0.074, SRMR = 0.058). See Supplementary S5 for all model parameters.

**Figure 3:**
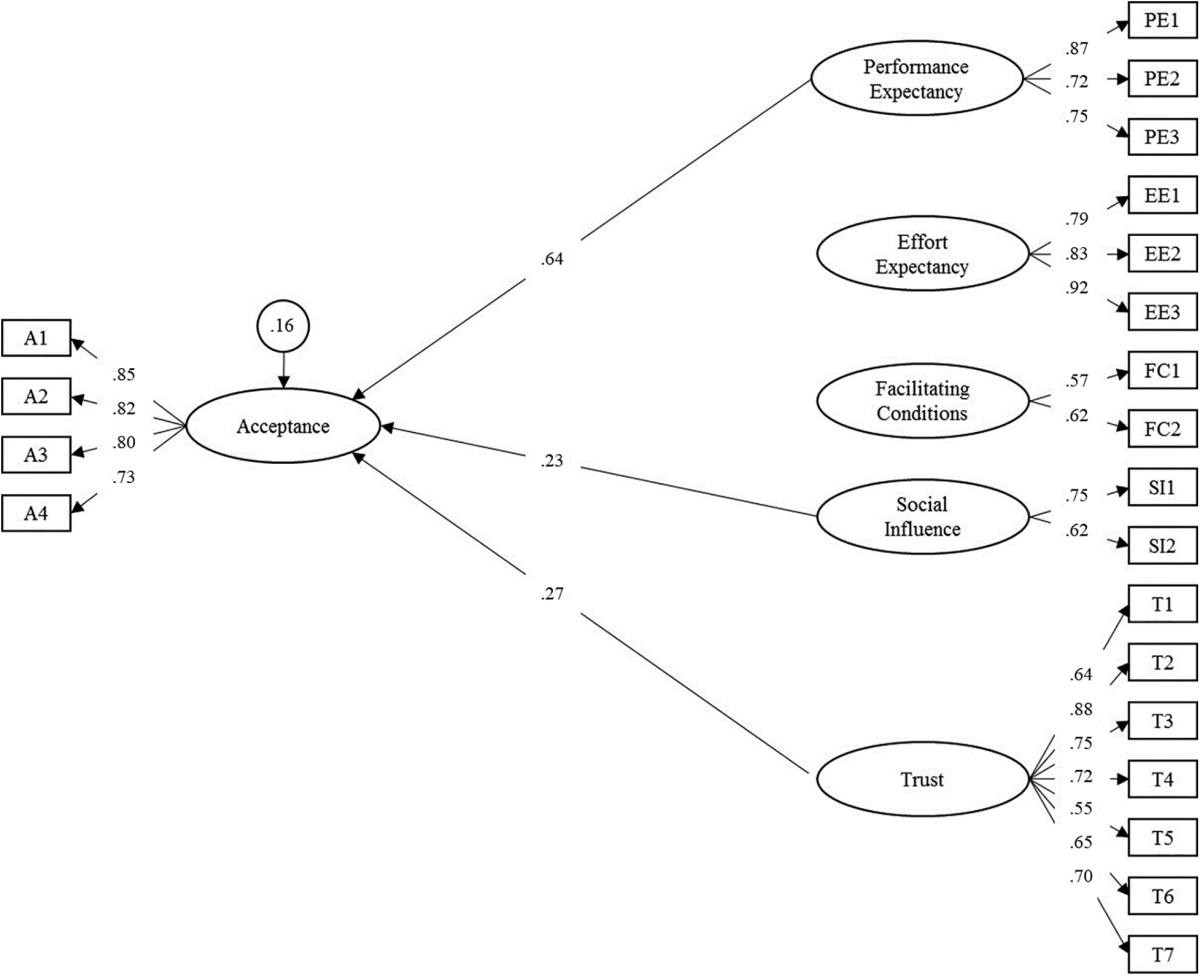
Adapted structural equation model for the acceptance towards smart sensing and associated predictor variables. Latent variables are represented in ellipses: A, acceptance; PE, performance expectancy; EE, effort expectancy; FC, facilitating conditions; SI, social influence; T, trust. Observed items are indicated as rectangles. Regression paths are represented by single-headed arrows. Non-significant paths were deleted in the final model. Residual variances of endogenous latent variables are presented in circles. All exogenous latent variables were allowed to correlate. For improved readability, all latent correlations and residual variances of manifest items were omitted. Please see Supplementary S6 for a full list of all parameters.

In the next step, the latent effects on acceptance across groups were analyzed. Performance expectancy (*γ *= 0.64, *p *< .001), social influence (*γ *= 0.23, *p *= .032) and trust (*γ *= 0.27, *p *= .039) were identified as predictor variables of acceptance (overall model fit: RMSEA = 0.073, SRMR = 0.059). Effort expectancy and facilitating conditions were not significant. The three variables explained 83.8% of the variance of the latent acceptance factor. The final path model is displayed in Figure 3. All model parameters are included in Supplementary S6.

### 3.3 Intervention effects

With an average self-reported acceptance of *M *= 13.47 (*SD *= 3.80, Min = 4, Max = 20) in the IG, the level of acceptance of smart sensing was not significantly higher than in the CG (*d *= 0.20, 95%-CI: −0.14–0.55, *t* = 1.17, *df* = 130, *p *= .244). This result was corroborated by the SEM analysis on latent level (*γ *= 0.25, 95%-CI: −0.16–0.65, *p *= .233). The distributions of acceptance levels in the IG and the CG are displayed in Figure 2.

In the IG, *n* = 26 (43.3%) participants stated interest to try smart sensing in a subsequent study (no interest: *n* = 20, 33.3%; not responded: *n* = 14, 23.3%). Among the 26 participants with interest, *n* = 12 (46.2%; 20.0% of all participants in the IG) actually installed the smart sensing app on their smartphones. The intervention effects and group-specific results are given in Table 2.

**Table 2:** Summary of intervention effects.

Outcome	CG	IG	Effect size	CI	*P*-value
Acceptance	12.64 (4.24)^a^	13.47 (3.80)^a^	*d* = 0.20^b^	−0.14–0.55	0.244
			*γ* = 0.25^c^	−0.16–0.65	0.233
Interest	*n* = 36 (50.0%)	*n* = 26 (43.3%)	OR* *= 0.76	0.38–1.52	0.445
Installation	*n* = 12 (16.7%)	*n* = 12 (20.0%)	OR* *= 1.13	0.47–2.73	0.777

CI, confidence interval; OR, odds ratio.

^a^ Mean (and standard deviation) of behavioral intention.

^b^ Mean difference between IG and CG based on observed data.

^c^ Unstandardized group difference between IG and CG based on SEM.

### 3.4 Sensitivity analysis

We found no significant correlation between education and behavioral intention (*r *= 0.06, *p *= .513). For personality only conscientiousness significantly correlated with behavioral intention (*r *= 0.22, *p *= .031). For the correlation matrix see Supplementary S7.

## 4 Discussion

We investigated the acceptance of smart sensing and the effect of an AFI towards smart sensing in a diabetes sample. The general acceptance toward smart sensing varied a lot between participants. The hypothetic model of acceptance towards smart sensing with three significant predictors (performance expectancy, social influence and trust) fit the data well, explained 84% variance of the self-reported acceptance, and thereby supported the validity of the model. The UTAUT-based intervention was not able to affect the acceptance of smart sensing.

Given that the treatment guidelines for diabetes recommend yearly and occasion-related diagnosis of common consequential and comorbid diseases including depression and other psychological disorders (81, 82), the integration of smart sensing systems, offering fine-granular, unobtrusive, objective and ecological valid assessments, could function as a form of passive screening support. This has the potential to improve healthcare systems, where resources are often restricted and time-efficient solutions are needed (23, 24). In order to translate encouraging findings from smart sensing studies (37, 38, 83) into tangible healthcare solutions, it is essential to address underlying processes governing both initial and long-term use. This involves a comprehensive understanding of user acceptance and the influencing factors. However, the challenge at hand appears to be two-fold. First, it necessitates a comprehensive examination of general acceptance, and second, it involves addressing the disparity between acceptance and the tangible use of the technology.

This study revealed an average baseline acceptance within our somatic sample (M = 12.64, SD = 4.24) which was higher compared to a healthy population (M = 10.9, SD = 3.73) (53). This inclination could be attributed to the pervasive presence of sensing and monitoring technologies in the daily routines of individuals living with diabetes (42). Although, this trend extended to the installation rates of smart sensing applications, a mere half of the study participants expressed interest in a smart sensing application. Moreover, only 17% and 20% of the CG and IG, respectively, proceeded to install the smart sensing app. Thus, the transfer from intention to use towards actual utilization needs to be addressed in future studies. This seems particularly relevant for the diabetes population, where existing daily self-management must be considered (84). Furthermore, it is important to not only measure the initiation of smart sensing usage but also its continuous use, including frequency and duration. Given that effective smart sensing systems often function as longitudinal assessments, it becomes crucial to explore strategies that foster optimal user performance. Within this context, user engagement and usability play a crucial role, warranting further investigation into personal habits that may facilitate the utilization of smart sensing (85, 86). Additionally, future research could additionally focus on design aspects supporting uptake of smart sensing applications (87, 88) as well specific factors such as potential structural or attitudinal barriers (89).

Consistently with a parallel study involving a healthy population (53), this study identified performance expectancy, social influence, and trust as associated factors of acceptance. Results regarding the hypothetic factor model of smart sensing acceptance and its potential determinants were consistent across these studies, supporting validity of the model. Notably, in our study, performance expectancy was of even greater importance in relation to other potential determinants. This could indicate the importance of addressing crucial needs and pointing out provided benefits of smart sensing.

In this study, the concept of trust was defined as the confidence that a system has the capability to assist in accomplishing an individual's objectives within situations characterized by uncertainty or vulnerability (52). With this understanding, people would show trust by believing in system predictions and following recommendations. However, given that privacy and data security are pivotal considerations in the implementation of smart sensing, future studies should delve into more nuanced distinctions within the realm of trust factors and explore diverse aspects. Smart sensing has the potential to facilitate highly sensitive health predictions, such as mental health screenings. Consequently, it would be beneficial to differentiate between trust in the system, trust in the potential predictions made by the system, and trust in the proper handling of this data. This distinction becomes particularly pertinent in light of political developments, underscoring the need for measures to ensure data privacy, security, and prevention of misuse (90). Moreover, the type of data collected and the entities with whom this data is shared play a crucial role in acceptance. For instance, in a recent study people indicated higher willingness to share sleep data with their physicians than location data, while the acceptance of the inclusion of this data in patient records was rather low in general (91). On a more general note, the public attitude and acceptance of digital health care systems remains a major barrier (53, 92–94). Broader public approaches could play a role in educating and thus leading to familiarization of digital healthcare on a population level. Moving forward, transparency concerning the usage, processing, and storage of data, as well as delineating who has access to the data and who does not, should be emphasized. This could be helpful to fostering user trust and, consequently, enhancing the acceptance of smart sensing technologies.

The implemented AFI did not impact acceptance of smart sensing. Therefore, a pivotal consideration is the improvement of the intervention itself. The current AFI format strategically targeted acceptance determinants based on the UTAUT, supplemented with everyday examples in a whiteboard design. Based on the cognitive theory of multimedia learning, dividing information into verbal and visual components results in reduced cognitive load (95). This could be further improved through a strengthened narrative approach, such as illustrating app functions and presenting extended case examples or short hands-on experience (95–97). Furthermore, expert opinions, as demonstrated in previous (53, 54, 56), might be useful to influence acceptance. It may be worthwhile to explore a mixed modality approach that includes expert opinions tailored to the specific needs and characteristics of the target population, alongside with dynamically visualized content. A similar study on acceptance of smart sensing in psychotherapy patients that focused on information presented by an expert showed promising results for an AFI to influence behavioral intention to use smart sensing (94). Future studies could additionally focus on the effectiveness of interventions in relation to baseline levels of acceptance. Given the above average acceptance in our sample a targeted approach towards individuals with lower levels of baseline acceptance could prove to be more beneficial. This could be implemented using a Solomon four-group study design and looking at interaction effects (98). Consequently, the effectiveness of AFI formats within the realm of smart sensing remains unclear and requires further exploration.

While interpreting the results and discussing future implications, it is crucial to acknowledge several limitations of the present research. First, the trial was critically underpowered to detect a significant intervention effect on acceptance, emphasizing the need for future confirmatory studies with an appropriately sized sample. Second, cross-sectional data are an insufficient basis for uncovering causal relationships. To comprehensively understand the dynamics of smart sensing acceptance and its potential determinants, longitudinal assessments featuring multiple measurement time points are needed. Third, the consideration of common-method bias is necessary, given that acceptance and its predicting variables were evaluated with the same questionnaire. This introduces a source of variance attributable to the measurement method rather than to the constructs, potentially inflating higher variable correlations. To mitigate this, further independent acceptance outcome measures should be considered. The generalization of our findings is further limited as our sample exhibited an overrepresentation of individuals with reported German nationality and high education levels. Moreover, despite covering a broad age range (27–81 years), the relatively high average age (58 years) poses a limitation on the transfer of the findings to a younger population. This is particularly pertinent given the elevated technological affinity and smartphone usage rates among younger individuals (99), potentially leading to distinct differences in the acceptance of smart sensing. Furthermore, the reported mental health symptoms, including depression, anxiety, and diabetes distress, were within a sub-clinical range. Given that performance expectancy emerged as the most strongly associated correlate of acceptance, it is plausible that the perceived personal benefit might be higher for more burdened individuals. These nuances should be taken into account in future research to provide a more comprehensive understanding of smart sensing acceptance across diverse contexts.

## 5 Conclusions

This study found a heterogenous distribution of acceptance of smart sensing with a relatively large percentage of participant reporting low acceptance, posing a hindrance to the implementation of smart sensing in research and practice. Performance expectancy, social influence, and trust in smart sensing were strongly associated with higher acceptance, suggesting that these aspects may constitute relevant influencing factors towards acceptance. Especially perceived benefit influenced the acceptance amongst the diabetic sample and should be paid special attention in the future. The developed AFI did not affect smart sensing acceptance, thus more effective intervention strategies must be developed. Further exploration of acceptance facilitating interventions on smart sensing are needed. Moving forward, research should look into barriers towards acceptance of smart sensing, which are essential for future implementation in routine health care. The results from this study of people with diabetes furthermore suggest that looking into different somatic areas and groups might detect important individual differences regarding smart sensing acceptance. To fully harness the potential of smart sensing technologies, acceptance, implementation and relevant stakeholders need to be taken into account.

## Data Availability

Data requests should be directed to the corresponding author (JK). Data can be shared with researchers who provide a methodologically sound proposal, which is not already covered by other researchers. Data can only be shared for projects if the General Data Protection Regulation is met. Requestors may need to sign additional data access agreements. Support depends on available resources.
