# Supplementary material for: Acceptance of smart sensing, its determinants, and the efficacy of an acceptance-facilitating intervention in people with diabetes: results from a randomized controlled trial
Source: Front Digit Health. 2024 May 28;6:1352762. doi: 10.3389/fdgth.2024.1352762 (PMC11165071; doi:10.3389/fdgth.2024.1352762)
Supplement: Supplementary file 1 [file Datasheet1.docx]

Supplementary Material

# Supplement 1 – CONSORT checklist.

| **Section/Topic** | **Item No** | **Checklist item** | **Reported on page No** |
| --- | --- | --- | --- |
| **Title and abstract** | | | |
|  | 1a | Identification as a randomised trial in the title | 1 |
|  | 1b | Structured summary of trial design, methods, results, and conclusions (for specific guidance see CONSORT for abstracts) | 1 |
| **Introduction** | | | |
| Background and objectives | 2a | Scientific background and explanation of rationale | 2 |
|  | 2b | Specific objectives or hypotheses | 2f |
| **Methods** | | | |
| Trial design | 3a | Description of trial design (such as parallel, factorial) including allocation ratio | 3 |
|  | 3b | Important changes to methods after trial commencement (such as eligibility criteria), with reasons | n.a. |
| Participants | 4a | Eligibility criteria for participants | 3 |
|  | 4b | Settings and locations where the data were collected | 3 |
| Interventions | 5 | The interventions for each group with sufficient details to allow replication, including how and when they were actually administered | 3 & Supplement 2 |
| Outcomes | 6a | Completely defined pre-specified primary and secondary outcome measures, including how and when they were assessed | 3f |
|  | 6b | Any changes to trial outcomes after the trial commenced, with reasons | n.a. |
| Sample size | 7a | How sample size was determined | n.a. |
|  | 7b | When applicable, explanation of any interim analyses and stopping guidelines | n.a. |
| Randomisation: |  |  |  |
| Sequence generation | 8a | Method used to generate the random allocation sequence | 3 |
|  | 8b | Type of randomisation; details of any restriction (such as blocking and block size) | 3 |
| Allocation concealment mechanism | 9 | Mechanism used to implement the random allocation sequence (such as sequentially numbered containers), describing any steps taken to conceal the sequence until interventions were assigned | n.a. |
| Implementation | 10 | Who generated the random allocation sequence, who enrolled participants, and who assigned participants to interventions | 3 |
| Blinding | 11a | If done, who was blinded after assignment to interventions (for example, participants, care providers, those assessing outcomes) and how | n.a. |
|  | 11b | If relevant, description of the similarity of interventions | n.a. |
| Statistical methods | 12a | Statistical methods used to compare groups for primary and secondary outcomes | 4 |
|  | 12b | Methods for additional analyses, such as subgroup analyses and adjusted analyses | 4 |
| **Results** | | | |
| Participant flow (a diagram is strongly recommended) | 13a | For each group, the numbers of participants who were randomly assigned, received intended treatment, and were analysed for the primary outcome | 5 |
|  | 13b | For each group, losses and exclusions after randomisation, together with reasons | 5 |
| Recruitment | 14a | Dates defining the periods of recruitment and follow-up | 3 |
|  | 14b | Why the trial ended or was stopped | n.a. |
| Baseline data | 15 | A table showing baseline demographic and clinical characteristics for each group | 5 |
| Numbers analysed | 16 | For each group, number of participants (denominator) included in each analysis and whether the analysis was by original assigned groups | 5 |
| Outcomes and estimation | 17a | For each primary and secondary outcome, results for each group, and the estimated effect size and its precision (such as 95% confidence interval) | 5f |
|  | 17b | For binary outcomes, presentation of both absolute and relative effect sizes is recommended | 5f |
| Ancillary analyses | 18 | Results of any other analyses performed, including subgroup analyses and adjusted analyses, distinguishing pre-specified from exploratory | 5f |
| Harms | 19 | All important harms or unintended effects in each group (for specific guidance see CONSORT for harms) | n.a. |
| **Discussion** | | | |
| Limitations | 20 | Trial limitations, addressing sources of potential bias, imprecision, and, if relevant, multiplicity of analyses | 8 |
| Generalisability | 21 | Generalisability (external validity, applicability) of the trial findings | 7f |
| Interpretation | 22 | Interpretation consistent with results, balancing benefits and harms, and considering other relevant evidence | 6f |
| **Other information** | | |  |
| Registration | 23 | Registration number and name of trial registry | n.a. |
| Protocol | 24 | Where the full trial protocol can be accessed, if available | n.a. |
| Funding | 25 | Sources of funding and other support (such as supply of drugs), role of funders | 9 |

# Supplement 2 - Summary of AFI components to target UTAUT factors and trust.

| Components | key concepts and examples in the AFI |
| --- | --- |
| performance expectancy (i.e., perceived personal benefit) | high burden by mental diseases and potential of smart sensing in assisted diagnosis, monitoring, early-recognition, personalized therapy;  two user reports and how they benefit:   - Sleep and physical activity monitoring improved sleep, fitness, and well-being by self-awareness, weight loss despite diabetes, - Monitoring of physical activity (i.e., step count) and assisted achievement of personal activity goals, improved weight, overall well-being, feelings of happiness and content;   information on research results and expected contribution of participation. |
| effort expectancy (i.e., expected ease of use) | passive collection of data by sensors,  optional integration of short active user inputs (i.e., short daily mood and well-being questionnaires) |
| social influence (i.e., perception others perceive the technology as use-worthy) | presentation of why others think smart sensing is use-worthy:   - information/examples provided by the researcher/expert - two reports by users |
| facilitating conditions (e.g., practical resources) | broad availability of smartphones and the necessary technology |
| trust | data security, anonymized and encrypted processing, consent to data collection methods |

*Note:* Due to copyright and agreements with the persons shown in the videos, the AFI is not publicly available. The here provided list provides a summary of the key concepts included in the acceptance facilitating intervention. More detailed information can be requested by the corresponding author.

# Supplement 3 - UTAUT items adapted to smart sensing.

| Variable | Items |
| --- | --- |
| Behavioral Intention | 1. I could imagine using a smart sensing app. 2. If offered, I would use a smart sensing app regularly 3. I would recommend a smart sensing app to a friend 4. I would be willing to pay for a smart sensing app |
| Performance Expectancy | 1. Using a smart sensing app could have positive effects on my health. 2. Using a smart sensing app and the collected data could help doctors in diagnoses 3. Overall, a smart sensing app could help managing health. |
| Effort  Expectancy | 1. Using a smart sensing app would be simple 2. Using a smart sensing app would be an easy task for me 3. A smart sensing app would be clear and easily comprehensible to me |
| Social  Influence | 1. People close to me would recommend me to use a smart sensing app 2. My general practitioner would recommend me to use a smart sensing app |
| Facilitating Conditions | 1. I have all necessary technical preconditions for using a smart sensing app 2. In case of technical problems with a smart sensing app I would receive technical support |
| *Note.* All items were presented in German and all present model fits refer to the German version of the questionnaire. Generalization to the here provided English items in pending. The German items can be requested from the corresponding author. | |

Items were based on: Terhorst, Y., Weilbacher, N., Suda, C., Simon, L., Messner, E.-M., Sander, L. B., & Baumeister, H. (2023). Acceptance of smart sensing: a barrier to implementation—results from a randomized controlled trial. In Frontiers in Digital Health (Vol. 5). Frontiers Media SA. https://doi.org/10.3389/fdgth.2023.1075266.

# Supplement 4 - R packages and versions

In the present study all analyses have been conducted in R. For an overview of all used packages and their respective versions please see below.


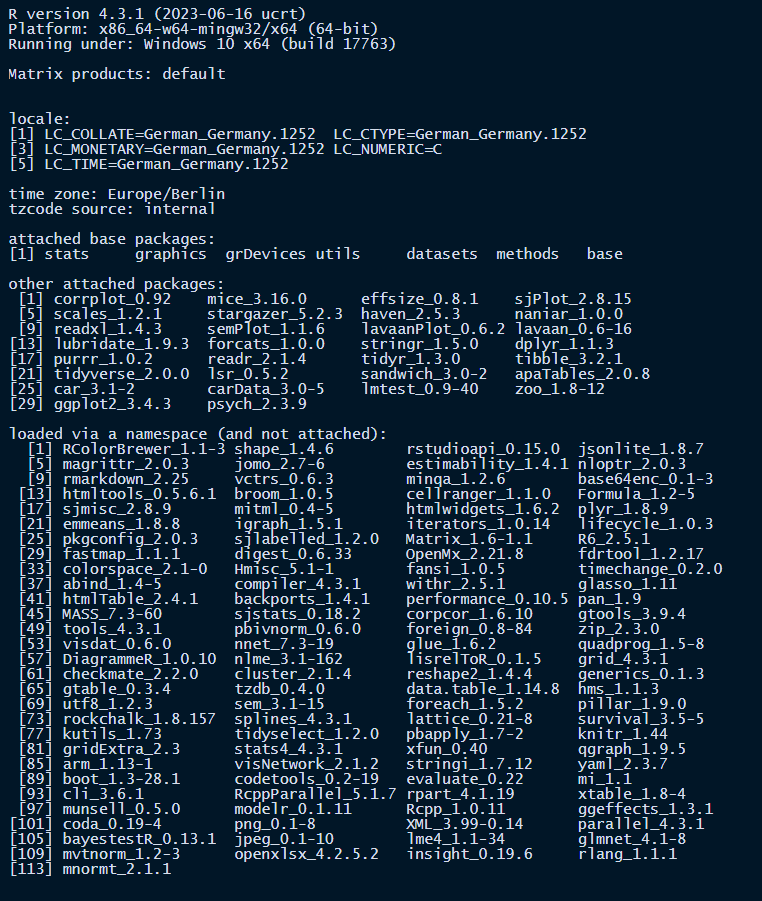


# Supplement 5 – Measurement model 1

A full list of all parameters in the measurement model (latent variables, covariances, and variances) can be found below. Acceptance is abbreviated (BI), performance expectancy (PE), effort expectancy (EE), facilitating conditions (FC), social influence (SI).

Modelfit: RMSEA=0.074, SRMR=0.058

Parameter Estimates:

Standard errors Sandwich

Information bread Observed

Observed information based on Hessian

Latent Variables:

Estimate Std.Err z-value P(>|z|) Std.lv Std.all

BI =~

UTAUT1.BI01. 1.000 1.065 0.857

UTAUT1.BI02. 0.935 0.056 16.803 0.000 0.996 0.816

UTAUT1.BI03. 0.759 0.061 12.539 0.000 0.808 0.804

UTAUT1.BI04. 0.802 0.085 9.461 0.000 0.854 0.734

PE =~

UTAUT1.PE01. 1.000 0.788 0.870

UTAUT1.PE02. 0.793 0.119 6.650 0.000 0.625 0.713

UTAUT1.PE03. 0.856 0.079 10.827 0.000 0.674 0.747

EE =~

UTAUT1.EE01. 1.000 0.765 0.793

UTAUT1.EE02. 1.098 0.198 5.538 0.000 0.841 0.829

UTAUT1.EE03. 1.228 0.131 9.348 0.000 0.940 0.915

FC =~

UTAUT4.FC01. 1.000 0.525 0.572

UTAUT4.FC02. 1.118 0.512 2.186 0.029 0.587 0.621

SI =~

UTAUT4.SI01. 1.000 0.630 0.756

UTAUT4.SI02. 0.907 0.315 2.881 0.004 0.571 0.613

TRUST =~

LETRASG.L1. 1.000 0.987 0.639

LETRASG.L2. 1.521 0.224 6.794 0.000 1.501 0.880

LETRASG.L3. 1.060 0.166 6.371 0.000 1.046 0.747

LETRASG.L4. 1.147 0.203 5.659 0.000 1.132 0.717

LETRASG.L5. 0.764 0.157 4.880 0.000 0.754 0.553

LETRASG.L6. 0.990 0.193 5.121 0.000 0.977 0.645

LETRASG.L7. 1.365 0.256 5.329 0.000 1.347 0.699

Covariances:

Estimate Std.Err z-value P(>|z|) Std.lv Std.all

.LETRASG.L5. ~~

.LETRASG.L6. 1.058 0.217 4.880 0.000 1.058 0.805

.UTAUT1.BI01. ~~

.UTAUT1.BI02. 0.285 0.088 3.252 0.001 0.285 0.630

.LETRASG.L4. ~~

.LETRASG.L6. 0.957 0.268 3.576 0.000 0.957 0.753

.LETRASG.L5. 0.771 0.232 3.329 0.001 0.771 0.617

BI ~~

PE 0.702 0.118 5.952 0.000 0.836 0.836

EE 0.569 0.112 5.106 0.000 0.699 0.699

FC 0.229 0.072 3.161 0.002 0.409 0.409

SI 0.352 0.113 3.128 0.002 0.526 0.526

TRUST 0.720 0.149 4.842 0.000 0.685 0.685

PE ~~

EE 0.464 0.105 4.436 0.000 0.770 0.770

FC 0.094 0.054 1.733 0.083 0.227 0.227

SI 0.137 0.099 1.392 0.164 0.276 0.276

TRUST 0.374 0.086 4.336 0.000 0.481 0.481

EE ~~

FC 0.244 0.069 3.547 0.000 0.608 0.608

SI 0.134 0.083 1.617 0.106 0.279 0.279

TRUST 0.423 0.100 4.247 0.000 0.560 0.560

FC ~~

SI 0.200 0.085 2.362 0.018 0.606 0.606

TRUST 0.225 0.099 2.265 0.024 0.433 0.433

SI ~~

TRUST 0.298 0.111 2.686 0.007 0.480 0.480

Intercepts:

Estimate Std.Err z-value P(>|z|) Std.lv Std.all

.UTAUT1.BI01. 3.659 0.108 33.845 0.000 3.659 2.946

.UTAUT1.BI02. 3.545 0.106 33.367 0.000 3.545 2.904

.UTAUT1.BI03. 3.258 0.087 37.252 0.000 3.258 3.242

.UTAUT1.BI04. 2.553 0.101 25.215 0.000 2.553 2.195

.UTAUT1.PE01. 3.811 0.079 48.342 0.000 3.811 4.208

.UTAUT1.PE02. 4.061 0.076 53.199 0.000 4.061 4.630

.UTAUT1.PE03. 3.705 0.079 47.168 0.000 3.705 4.105

.UTAUT1.EE01. 3.712 0.084 44.171 0.000 3.712 3.845

.UTAUT1.EE02. 3.902 0.088 44.206 0.000 3.902 3.848

.UTAUT1.EE03. 3.886 0.089 43.466 0.000 3.886 3.783

.UTAUT4.FC01. 3.599 0.081 44.411 0.000 3.599 3.920

.UTAUT4.FC02. 4.265 0.084 50.606 0.000 4.265 4.509

.UTAUT4.SI01. 3.041 0.073 41.422 0.000 3.041 3.649

.UTAUT4.SI02. 3.243 0.082 39.614 0.000 3.243 3.481

.LETRASG.L1. 4.903 0.136 36.102 0.000 4.903 3.173

.LETRASG.L2. 4.702 0.150 31.342 0.000 4.702 2.754

.LETRASG.L3. 5.574 0.124 45.070 0.000 5.574 3.983

.LETRASG.L4. 4.636 0.139 33.427 0.000 4.636 2.938

.LETRASG.L5. 4.664 0.120 38.887 0.000 4.664 3.420

.LETRASG.L6. 4.513 0.133 33.892 0.000 4.513 2.980

.LETRASG.L7. 4.235 0.169 25.034 0.000 4.235 2.198

BI 0.000 0.000 0.000

PE 0.000 0.000 0.000

EE 0.000 0.000 0.000

FC 0.000 0.000 0.000

SI 0.000 0.000 0.000

TRUST 0.000 0.000 0.000

Variances:

Estimate Std.Err z-value P(>|z|) Std.lv Std.all

.UTAUT1.BI01. 0.409 0.092 4.455 0.000 0.409 0.265

.UTAUT1.BI02. 0.499 0.097 5.122 0.000 0.499 0.335

.UTAUT1.BI03. 0.357 0.058 6.172 0.000 0.357 0.353

.UTAUT1.BI04. 0.624 0.104 6.004 0.000 0.624 0.461

.UTAUT1.PE01. 0.199 0.051 3.905 0.000 0.199 0.243

.UTAUT1.PE02. 0.378 0.065 5.828 0.000 0.378 0.492

.UTAUT1.PE03. 0.360 0.089 4.039 0.000 0.360 0.442

.UTAUT1.EE01. 0.346 0.079 4.361 0.000 0.346 0.371

.UTAUT1.EE02. 0.321 0.120 2.686 0.007 0.321 0.312

.UTAUT1.EE03. 0.172 0.049 3.505 0.000 0.172 0.163

.UTAUT4.FC01. 0.568 0.117 4.859 0.000 0.568 0.673

.UTAUT4.FC02. 0.550 0.154 3.577 0.000 0.550 0.615

.UTAUT4.SI01. 0.298 0.110 2.700 0.007 0.298 0.429

.UTAUT4.SI02. 0.542 0.164 3.304 0.001 0.542 0.624

.LETRASG.L1. 1.414 0.341 4.144 0.000 1.414 0.592

.LETRASG.L2. 0.660 0.172 3.843 0.000 0.660 0.226

.LETRASG.L3. 0.864 0.163 5.286 0.000 0.864 0.441

.LETRASG.L4. 1.209 0.285 4.235 0.000 1.209 0.486

.LETRASG.L5. 1.291 0.218 5.924 0.000 1.291 0.694

.LETRASG.L6. 1.338 0.246 5.438 0.000 1.338 0.583

.LETRASG.L7. 1.900 0.376 5.046 0.000 1.900 0.512

BI 1.134 0.166 6.837 0.000 1.000 1.000

PE 0.621 0.122 5.088 0.000 1.000 1.000

EE 0.586 0.147 3.999 0.000 1.000 1.000

FC 0.275 0.153 1.797 0.072 1.000 1.000

SI 0.396 0.142 2.788 0.005 1.000 1.000

TRUST 0.974 0.272 3.577 0.000 1.000 1.000

# Supplement 6 – Measurement model 2

A full list of all parameters in the measurement model (latent variables, covariances, and variances) can be found below. Acceptance is abbreviated (BI), performance expectancy (PE), effort expectancy (EE), facilitating conditions (FC), social influence (SI).

Modelfit: RMSEA=0.073, SRMR=0.059

Parameter Estimates:

Standard errors Sandwich

Information bread Observed

Observed information based on Hessian

Latent Variables:

Estimate Std.Err z-value P(>|z|) Std.lv Std.all

BI =~

UTAUT1.BI01. 1.000 1.065 0.858

UTAUT1.BI02. 0.935 0.056 16.761 0.000 0.996 0.816

UTAUT1.BI03. 0.758 0.060 12.720 0.000 0.808 0.804

UTAUT1.BI04. 0.801 0.083 9.697 0.000 0.853 0.734

PE =~

UTAUT1.PE01. 1.000 0.789 0.872

UTAUT1.PE02. 0.794 0.120 6.631 0.000 0.627 0.715

UTAUT1.PE03. 0.854 0.080 10.733 0.000 0.674 0.747

EE =~

UTAUT1.EE01. 1.000 0.765 0.793

UTAUT1.EE02. 1.098 0.197 5.559 0.000 0.840 0.829

UTAUT1.EE03. 1.229 0.131 9.371 0.000 0.940 0.916

FC =~

UTAUT4.FC01. 1.000 0.529 0.576

UTAUT4.FC02. 1.100 0.475 2.318 0.020 0.582 0.616

SI =~

UTAUT4.SI01. 1.000 0.623 0.747

UTAUT4.SI02. 0.923 0.293 3.152 0.002 0.575 0.617

TRUST =~

LETRASG.L1. 1.000 0.987 0.639

LETRASG.L2. 1.520 0.224 6.798 0.000 1.501 0.879

LETRASG.L3. 1.060 0.166 6.396 0.000 1.047 0.748

LETRASG.L4. 1.147 0.202 5.686 0.000 1.133 0.718

LETRASG.L5. 0.764 0.156 4.910 0.000 0.754 0.553

LETRASG.L6. 0.991 0.193 5.139 0.000 0.978 0.646

LETRASG.L7. 1.364 0.256 5.331 0.000 1.347 0.699

Regressions:

Estimate Std.Err z-value P(>|z|) Std.lv Std.all

BI ~

PE 0.867 0.119 7.266 0.000 0.642 0.642

SI 0.385 0.208 1.849 0.032 0.225 0.225

TRUST 0.289 0.165 1.756 0.040 0.268 0.268

Covariances:

Estimate Std.Err z-value P(>|z|) Std.lv Std.all

.LETRASG.L5. ~~

.LETRASG.L6. 1.057 0.217 4.875 0.000 1.057 0.805

.UTAUT1.BI01. ~~

.UTAUT1.BI02. 0.284 0.088 3.224 0.001 0.284 0.630

.LETRASG.L4. ~~

.LETRASG.L6. 0.956 0.266 3.594 0.000 0.956 0.753

.LETRASG.L5. 0.770 0.232 3.326 0.001 0.770 0.617

PE ~~

EE 0.463 0.104 4.437 0.000 0.766 0.766

FC 0.095 0.056 1.685 0.092 0.227 0.227

SI 0.135 0.095 1.417 0.157 0.275 0.275

TRUST 0.375 0.086 4.333 0.000 0.481 0.481

EE ~~

FC 0.246 0.067 3.660 0.000 0.607 0.607

SI 0.131 0.081 1.624 0.104 0.276 0.276

TRUST 0.422 0.099 4.268 0.000 0.558 0.558

FC ~~

SI 0.202 0.081 2.495 0.013 0.614 0.614

TRUST 0.228 0.094 2.435 0.015 0.436 0.436

SI ~~

TRUST 0.294 0.107 2.763 0.006 0.479 0.479

Intercepts:

Estimate Std.Err z-value P(>|z|) Std.lv Std.all

.UTAUT1.BI01. 3.659 0.108 33.845 0.000 3.659 2.946

.UTAUT1.BI02. 3.545 0.106 33.367 0.000 3.545 2.904

.UTAUT1.BI03. 3.258 0.087 37.252 0.000 3.258 3.242

.UTAUT1.BI04. 2.553 0.101 25.215 0.000 2.553 2.195

.UTAUT1.PE01. 3.811 0.079 48.342 0.000 3.811 4.208

.UTAUT1.PE02. 4.061 0.076 53.198 0.000 4.061 4.630

.UTAUT1.PE03. 3.705 0.079 47.168 0.000 3.705 4.105

.UTAUT1.EE01. 3.712 0.084 44.171 0.000 3.712 3.845

.UTAUT1.EE02. 3.902 0.088 44.206 0.000 3.902 3.848

.UTAUT1.EE03. 3.886 0.089 43.466 0.000 3.886 3.783

.UTAUT4.FC01. 3.599 0.081 44.423 0.000 3.599 3.919

.UTAUT4.FC02. 4.265 0.084 50.659 0.000 4.265 4.509

.UTAUT4.SI01. 3.041 0.073 41.419 0.000 3.041 3.649

.UTAUT4.SI02. 3.243 0.082 39.593 0.000 3.243 3.481

.LETRASG.L1. 4.903 0.136 36.104 0.000 4.903 3.173

.LETRASG.L2. 4.702 0.150 31.360 0.000 4.702 2.754

.LETRASG.L3. 5.575 0.124 45.079 0.000 5.575 3.983

.LETRASG.L4. 4.636 0.139 33.435 0.000 4.636 2.938

.LETRASG.L5. 4.664 0.120 38.886 0.000 4.664 3.420

.LETRASG.L6. 4.513 0.133 33.901 0.000 4.513 2.980

.LETRASG.L7. 4.235 0.169 25.049 0.000 4.235 2.198

.BI 0.000 0.000 0.000

PE 0.000 0.000 0.000

EE 0.000 0.000 0.000

FC 0.000 0.000 0.000

SI 0.000 0.000 0.000

TRUST 0.000 0.000 0.000

Variances:

Estimate Std.Err z-value P(>|z|) Std.lv Std.all

.UTAUT1.BI01. 0.408 0.092 4.431 0.000 0.408 0.264

.UTAUT1.BI02. 0.498 0.098 5.073 0.000 0.498 0.334

.UTAUT1.BI03. 0.357 0.058 6.157 0.000 0.357 0.354

.UTAUT1.BI04. 0.625 0.101 6.208 0.000 0.625 0.462

.UTAUT1.PE01. 0.197 0.048 4.079 0.000 0.197 0.240

.UTAUT1.PE02. 0.376 0.064 5.873 0.000 0.376 0.489

.UTAUT1.PE03. 0.359 0.089 4.051 0.000 0.359 0.442

.UTAUT1.EE01. 0.346 0.079 4.382 0.000 0.346 0.372

.UTAUT1.EE02. 0.322 0.119 2.700 0.007 0.322 0.313

.UTAUT1.EE03. 0.171 0.049 3.466 0.001 0.171 0.162

.UTAUT4.FC01. 0.563 0.113 4.990 0.000 0.563 0.668

.UTAUT4.FC02. 0.556 0.145 3.830 0.000 0.556 0.621

.UTAUT4.SI01. 0.307 0.100 3.075 0.002 0.307 0.442

.UTAUT4.SI02. 0.537 0.158 3.391 0.001 0.537 0.619

.LETRASG.L1. 1.414 0.342 4.139 0.000 1.414 0.592

.LETRASG.L2. 0.662 0.173 3.829 0.000 0.662 0.227

.LETRASG.L3. 0.863 0.162 5.331 0.000 0.863 0.441

.LETRASG.L4. 1.208 0.283 4.262 0.000 1.208 0.485

.LETRASG.L5. 1.291 0.218 5.923 0.000 1.291 0.694

.LETRASG.L6. 1.337 0.245 5.462 0.000 1.337 0.583

.LETRASG.L7. 1.900 0.377 5.043 0.000 1.900 0.512

.BI 0.184 0.087 2.125 0.034 0.162 0.162

PE 0.623 0.122 5.111 0.000 1.000 1.000

EE 0.586 0.146 4.015 0.000 1.000 1.000

FC 0.280 0.152 1.846 0.065 1.000 1.000

SI 0.388 0.131 2.965 0.003 1.000 1.000

TRUST 0.975 0.272 3.585 0.000 1.000 1.000

# Supplement 7 – Correlation Matrix between Acceptance, Education and Personality

|  | | | | | |  |  |
| --- | --- | --- | --- | --- | --- | --- | --- |
|  | Acceptance | Education | Extraversion | Agreeableness | Conscientious-ness | | Neuroticism |
| Education | .06 | - |  |  |  | |  |
| Extraversion | .10 | .07 | - |  |  | |  |
| Agreeableness | .22* | .00 | -.14 | - |  | |  |
| Conscientiousness | .19 | -.10 | .00 | .07 | - | |  |
| Neuroticism | -.01 | -.12 | -.16 | -.14 | .58** | | - |
| Openness | .08 | .02 | .18 | .05 | .10 | | .06 |
| *Note*. * indicates p < .05, and ** indicates p < .01. | | | | | |  |  |
